# Editorial: New concepts in revascularization strategies and antithrombotic therapies in patients with non-ST elevated acute coronary syndromes

**DOI:** 10.3389/fcvm.2025.1736781

**Published:** 2025-11-24

**Authors:** Marco Mojoli, Krishnaraj Rathod, Marco Zuin

**Affiliations:** 1 Division of Cardiology, Azienda Sanitaria Friuli Occidentale (ASFO), Ospedale Santa Maria Degli Angeli, Pordenone, Italy; 2 Barts Heart Centre, St. Bartholomew’s Hospital, and William Harvey Research Institute, Queen Mary University of London, London, United Kingdom; 3 Department of Translational Medicine, University of Ferrara, Ferrara, Italy; 4 Department of Cardio-Thoraco-Vascular Sciences and Public Health, University of Padova, Padua, Italy; 5 Department of Cardiology, Madre Teresa di Calcutta Hospital, AULSS 6, Ospedali Riuniti Padova Sud, Monselice, Italy

**Keywords:** NSTE-ACS, PCI, percutaneous coronary intervention, CABG (coronary arterial bypass graft), myocardial infarction, myocardial revascularization, antithrombotic therapy

Non-ST-elevation acute coronary syndromes (NSTE-ACS) represent a complex and heterogeneous clinical entity in contemporary cardiovascular medicine. Compared with patients presenting with ST-elevation myocardial infarction (STEMI), those with NSTE-ACS are typically older and burdened with a higher prevalence of comorbidities. An intricate interplay between ischemic and bleeding risks, common to both acute and chronic coronary syndromes, further interacts with age and sex (1), underscoring the need for individualized treatment approaches. Despite substantial advances in interventional techniques and pharmacologic treatments (2), the optimal revascularization strategy, timing, and antithrombotic regimen remain matter of investigation and debate.

This Research Topic, “*New Concepts in Revascularization Strategies and Antithrombotic Therapies in Patients With Non-ST Elevated Acute Coronary Syndromes (NSTE-ACS)*,” brings together several timely contributions that collectively advance the understanding of coronary revascularization decision-making, functional assessment, and precision antithrombotic care in this high-risk population.

A central theme emerging from this collection is the continuous refinement of revascularization strategies in patients with NSTE-ACS. In their comprehensive review, Zuccarelli et al. examined contemporary evidence comparing percutaneous coronary intervention (PCI) and coronary artery bypass grafting (CABG) in non–ST-elevation myocardial infarction (NSTEMI). Their analysis emphasizes that the choice between PCI and CABG must remain individualized, guided by anatomical complexity, comorbid burden, and procedural feasibility. Importantly, the authors highlight the key role of the multidisciplinary Heart Team in tailoring revascularization to patient-specific anatomy and hemodynamic profile. While PCI remains less invasive and enables faster revascularization, CABG continues to offer superior long-term freedom from myocardial infarction and repeat revascularization, in patients with more complex multivessel or left main disease who are suitable for surgery.

Further contributing to patient stratification, Sun et al. introduced the SAVE risk score, a novel and promising tool for early identification of NSTE-ACS patients with a totally occluded infarct-related artery (IRA-TOCA). Their retrospective analysis revealed that these patients represent a distinct high-risk subgroup characterized by greater post-procedural troponin release and adverse outcomes. The SAVE score outperformed the conventional GRACE score in identifying such cases, suggesting potential utility for early triage and prioritization of an invasive procedure. This study exemplifies the growing movement toward precision-based risk stratification to guide revascularization timing and resource allocation in NSTE-ACS care.

Beyond revascularization, optimization of antithrombotic therapy represents another cornerstone of managment. Li et al. conducted a systematic review and meta-analysis comparing ticagrelor vs. clopidogrel-based dual antiplatelet therapy (DAPT) in patients with acute coronary syndrome complicated by chronic kidney disease (CKD). Their results suggest that ticagrelor-based regimens significantly reduced the risk of major adverse cardiovascular events and stroke without a significant increase in major bleeding. Moreover, shortened DAPT duration did not confer any additional benefit. These findings reinforce current trends toward individualized DAPT tailoring, balancing ischemic and bleeding risks, especially in high-risk subgroups such as those with CKD, in whom both thrombotic and hemorrhagic risks are heightened.

In a complement to these clinical perspectives, Yan et al. explored innovative strategies to enhance microvascular perfusion in the setting of myocardial ischemia and reperfusion. Although primarily focused on STEMI, their discussion of intracoronary thrombolysis combined with thrombus aspiration holds conceptual relevance for patients undergoing PCI in the presence of a thrombotic lesion. Persistent microvascular obstruction (MVO) remains a major determinant of suboptimal reperfusion and adverse ventricular remodeling even after successful epicardial revascularization. By revisiting the potential role of localized thrombolytic therapy, this review revives interest in adjunctive techniques that may inform future interventional protocols.

Finally, Vassilev et al. presented a practical advance in the field of coronary functional assessment. Their brief report proposed an arrhythmia prevention protocol combining intravenous lidocaine pretreatment with intracoronary papaverine administration during invasive physiological testing. The study evidenced that lidocaine substantially mitigates the incidence of papaverine-induced ventricular arrhythmias while maintaining reliable hyperemia for fractional flow reserve (FFR), coronary flow reserve (CFR), and index of microcirculatory resistance (IMR) measurements. This innovation enhances procedural safety and may encourage broader clinical adoption of invasive physiological assessment in both chronic and acute coronary syndromes.

Taken together, the contributions within this Research Topic exemplify the multidimensional evolution of NSTE-ACS management. The integration of refined risk stratification (SAVE score), individualized revascularization planning (Heart Team–guided PCI vs. CABG), tailored antithrombotic therapy (ticagrelor in CKD), and precision physiological assessment (lidocaine–papaverine protocol) reflects the convergence of clinical evidence, technology, and systems-based care. Collectively, these studies reinforce a paradigm shift from “one-size-fits-all” strategies toward personalized, data-driven decision-making that harmonized ischemic protection, bleeding safety and procedural efficacy.

The Editors hope that this Research Topic will inspire further research into the interplay between coronary pathophysiology, procedural strategy, and systemic therapy, ultimately improving the outcomes of patients with NSTE-ACS.
